# Effect of Multi-Pass Friction Stir Processing on Mechanical Properties for AA2024/Al_2_O_3_ Nanocomposites

**DOI:** 10.3390/ma10091053

**Published:** 2017-09-08

**Authors:** Essam Moustafa

**Affiliations:** Mechanical Engineering Department, Faculty of Engineering, King Abdulaziz University, Jeddah 21589, Saudi Arabia; abmostafa@kau.edu.sa; Tel.: +966-540886498

**Keywords:** FSP, multi-pass, nanoparticles, surface, composite, reinforcement, hardness, MMNCs

## Abstract

In the present work, an aluminum metal matrix reinforced with (Al_2_O_3_) nanoparticles was fabricated as a surface composite sheet using friction stir processing (FSP). The effects of processing parameters on mechanical properties, hardness, and microstructure grain were investigated. The results revealed that multi-pass FSP causes a homogeneous distribution and good dispersion of Al_2_O_3_ in the metal matrix, and consequently an increase in the hardness of the matrix composites. A finer grain is observed in the microstructure examination in specimens subjected to second and third passes of FSP. The improvement in the grain refinement is 80% compared to base metal. The processing parameters, particularly rotational tool speed and pass number in FSP, have a major effect on strength properties and surface hardness. The ultimate tensile strength (UTS) and the average hardness are improved by 25% and 46%, respectively, due to presence of reinforcement Al_2_O_3_ nanoparticles.

## 1. Introduction

Recently, friction stir processing (FSP), and other processes known as surface modification methods have been the focus of much attention from researchers. As a result of intensive plastic deformation during FSP, the microstructure in the processed zone changed significantly [1,2]. The effect of process parameters on the mechanical properties and the quality of processed material has been investigated in [3,4,5,6,7,8,9,10,11], where it was reported that the quality of surfaces subjected to FSP was influenced by rotation speed. The tool rotation speed has a significant influence on mechanical properties and microstructure grain refinement as a result of increasing the heat input causes softening and intense stirring action of the rotating tool pin. This causes better grain refinement, which leads to an improvement in the mechanical properties [12,13,14,15]. At lower travel speeds, the processed zone exposed to higher temperature due to increasing stirred time results in a grain size decrease in composites, with a better dispersion of reinforcement particles [16]. The effects of multi-pass FSP on the microstructure, microhardness, and tensile strength of the metal alloy and composites have been studied by many investigators, but there was a disparity in the results with respect to the difference in the materials used. The importance of these additional passes lies in the improvement of the mechanical properties and the fine and homogenous dispersion of the reinforcement particles in the metal matrix composites.

There is variation in the results of the hardness tests among many researchers; some studies deal with pure alloys without any additives, and they conclude that the hardness of the processed zone was decreased by increasing the number of FSP passes [17,18]. On the other hand, in studies using reinforcement particles in the matrix, it was revealed that the hardness was increased by the increasing number of passes compared to as-received metal [19,20,21]. An even number of FSP multi-passes was performed in order to improve the mechanical properties of the in situ Cu/Sic composite. The authors reasoned that this improvement because of reduced porosity content and enhanced bonding between the copper matrix and Sic particles [21]. Liu et al. [22] observed that as the number of FSP passes increased, there was good dispersion of CNT in the matrix, and the maximum tensile strength improved with increasing pass numbers. FSP for the AA7075 alloy using multiple passes demonstrated that the largest strain rate was obtained at a single pass [23]. The dynamic recrystallization in the processed zone and accumulated heat, due to increasing the number of passes in FSP, led to increasing equiaxed grain sizes with high-angle grain boundaries [24]. Multi-pass FSP has a great effect on the refinement of grain size.

On studying the effect of overlap multi-pass FSP on the hypereutectic Al–30Si alloy, the investigators reported that increasing the FSP passes decreased the corrosion rate of alloy due to the reduction of grain and silicon particle sizes as well as an increase in the homogeneity of the microstructure [25]. Sarkari Khorrami et al. [26] revealed that the increase in FSP pass number did not have significant effects on the stirring zone of the specimen subjected to FSP without reinforcement nanoparticles. In contrast, FSP with Sic nanoparticles can intensely affect the distribution of the nanoparticles and the microstructural evolution and mechanical properties of the processed zone. A surface composite matrix using FSP was affected by the type of reinforced particles and methods of inserting these particles into the alloys during processing. FSP is considered one of the major techniques used in fabricating surface composites, and the results showed that as the number of FSP passes increases it causes a uniform dispersion of reinforced particles [27,28,29,30,31,32]. Three common methods for inserting reinforcement particles in the fabrication composite matrix are by grooves, drilled holes, and by using cover plate, as specified in one study [26].

The current study uses Al_2_O_3_ nanoparticles as reinforcement particles with the AA 2014 aluminum alloy in order to fabricate a surface composite metal matrix using multiple FSP. The aim of this work is to study the effect of multiple-pass FSP on the mechanical properties and grain microstructure. Furthermore, the effect of Al_2_O_3_ nanoparticles on the hardness and the tensile strength of the matrix is studied. Moreover, this work focuses on the nanocomposite matrix fabricated by multi-pass FSP and the effect of regular and perfect distribution of nanoparticles on the quality of the composite matrix.

## 2. Materials and Methods

### 2.1. Material and Processing

**Figure 1:**
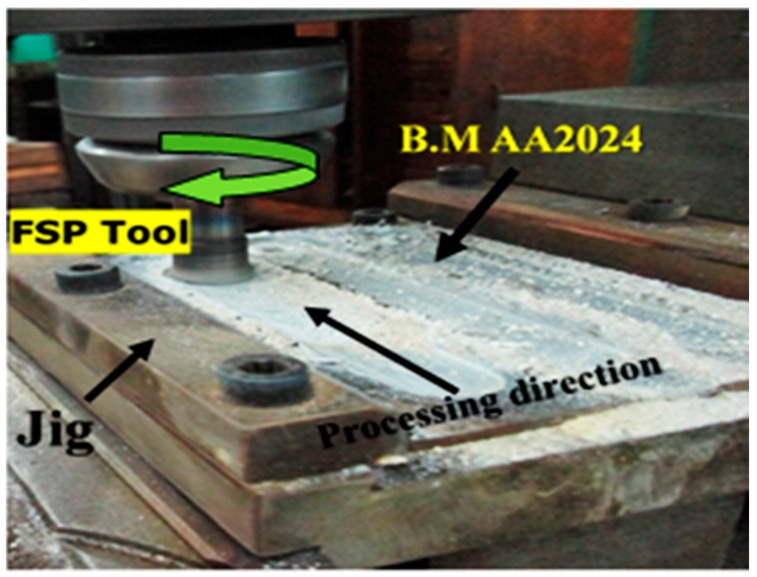
Friction stir processing (FSP) using an automatic vertical milling machine.

The AA 2024 wrought alloy with a thickness of 3.5 mm has a nominal composition as listed in the Table 1, and was subjected to FSP. Al_2_O_3_ nanoparticles with an average diameter 30 nm have been used to reinforce aluminum metal matrix nanocomposites (Al MMNCs). The base metal sheets were prepared and machined for suitable size. The plates were grooved with 3 mm width and 2 mm depth before application of FSP. A hardened K-110 tool steel was used as a tool for FSP. Tool geometry was designed based on previous studies [33,34]. Furthermore, there are several attempts were performed on the tool pin and shoulder diameters to process succeeds. The tool was machined with a cylindrical shape with a shoulder diameter of (Ø25 mm), a pin diameter of (Ø8 mm) and tool pin has a 2.5 mm length. An automatic vertical milling machine was used for processing, as shown in Figure 1. The tool rotation speed varied from 900, 1120, 1400, to 1800 rpm with three travel speeds of 10, 15, and 20 mm/min. A tool tilt angle of 2 degrees was used. Tool plunge speed (0.5 mm/s) and dwell time (2 s) were kept constant. Three multi-passes were performed in the processing.

### 2.2. Tension Test

**Figure 2:**
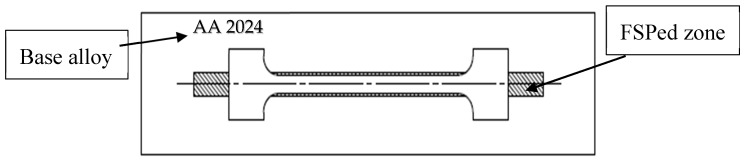
Schematic drawing of the tension specimen and direction of cutting.

**Figure 3:**
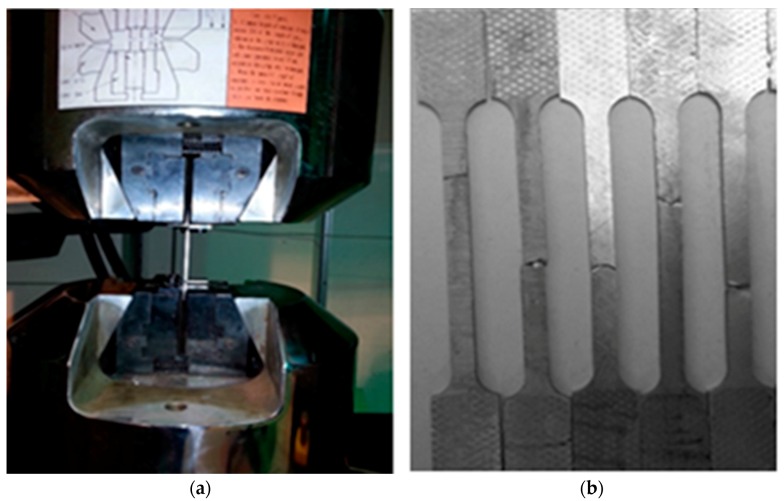
Tension test (**a**) Universal tension test machine; (**b**) samples after the applied tensile test.

A standard specimen was prepared according to the ASTM *Standard Test Methods for Tension Testing Wrought and Cast Aluminum- and Magnesium-Alloy Products* (ASTM B557) in tension testing for wrought and cast aluminum alloys. The specimens were cut parallel to the friction stir-processed direction as shown in Figure 2, using a CNC milling machine. The tensile tests were performed using the MTS tension machine, as shown in Figure 3. Applied load and extension were recorded for each tested specimen. The calculation of Young’s modulus, yield strength, ultimate tensile strength, and elongation were evaluated.

### 2.3. Microstructure and SEM Analysis

With respect to specimen preparation for microstructure and microhardness tests, in order to identify and evaluate the microstructure of the MMNCs, the specimens were prepared properly through basic steps including:

Sectioning and cutting: The specimens for metallographic examination were sectioned perpendicular to the processing direction, and cut into the required sizes in each test. Grinding and polishing: Specimens were ground progressively under water. Each stage progressively removes and replaces the larger surface scratches with smaller ones using silicon carbide abrasive discs with different grades. Then, they were polished using 10 μm alumina paste and 3 μm diamond paste. Etching: The samples were etched with classical Keller’s reagent (2 mL HF (48%) + 6 mL HNO_3_ + 91 mL distilled water) at room temperature for a few seconds to reveal the macrostructure and microstructure of the processed samples until the desired contrast was obtained.

The microstructural analysis was conducted on the friction stir-processed specimens to study the effect of process parameters on grain structure and the distribution of the Al_2_O_3_ particles in the matrix. Microstructural characteristics of FSP specimens were investigated using an optical metallurgical microscope GX41. The samples were cut in the stirred zone and analyzed using a scanning electron microscope (SEM), which was equipped with energy-dispersive X-ray spectroscopy (EDS). The desired samples were prepared using sputter coater before being submitted to SEM. The aim of this stage is to enable the scanning electron microscope to display all the desired particles clearly. This test has been carried out to examine the nanopowder grain size, volume fraction for Al_2_O_3_ particles and their distribution inside MMNCs, and for scanning of the samples after tension tests in order to examine the fracture type.

### 2.4. Microhardness Testing

A Vicker’s microhardness testing machine was used to measure the hardness across the processed region with a 1 kg load with a 10 s dwell time according to ASTM E-384-05 guidelines. Eight readings were taken at close proximity in each zone and mean values were considered for further analysis and discussion. The samples subjected to the microhardness test were surface-cleaned and polished. Furthermore, the indenter measured eight readings along the sample surface using steps of 2 mm.

## 3. Results and Analysis

### 3.1. Effect of Processing Parameters on the Tensile Strength

**Figure 4:**
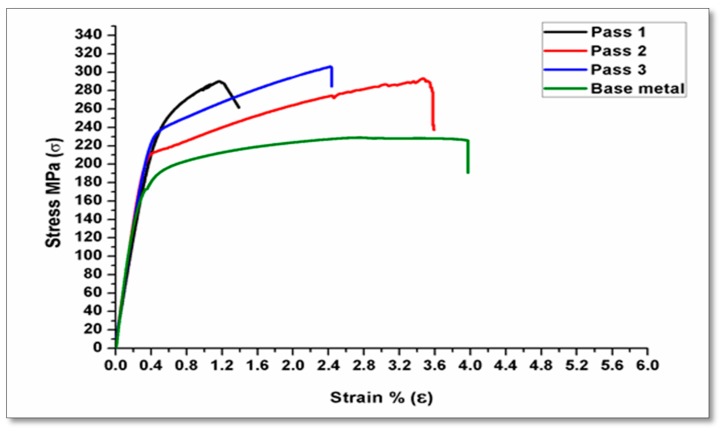
Effect of pass number on tensile strength at 900 rpm with a 15 mm/min travel speed.

**Figure 5:**
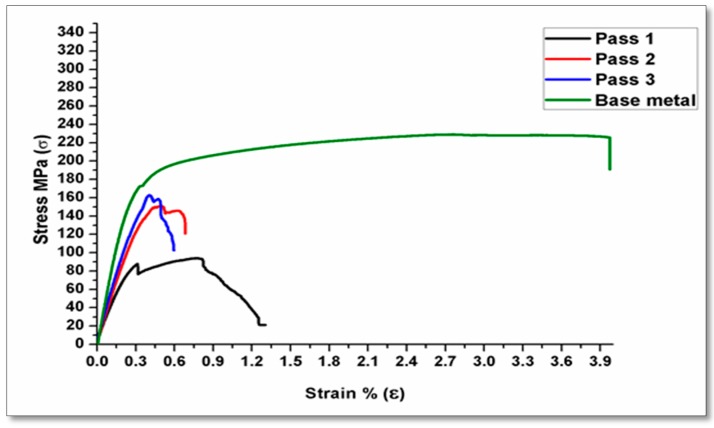
Effect of pass number on tensile strength at 1800 rpm with a 15 mm/min travel speed.

In FSP, the tool rotation speed is considered the most significant process variable, which in turn increases the material temperature and the stirring action, causing mixing of material and nanoparticles around the rotating pin. The four-tool rotation speeds (ω) were carried out at 900, 1120, 1400, and 1800 rpm. The processing speed rates are executed by three linear speeds (ν) at 10, 15, and 20 mm/min. The tensile strengths for the specimens subjected to FSP were improved by increasing the rotation speed until reaching a specific limit as shown in Figure 4. The rotation speed of 900 rpm produced a good tensile strength. On the other hand, Figure 5, represents the tensile curve of the executed processing at 1800 rpm at the same travel speed. It shows a lower tensile strength, and many researchers [35,36,37] have obtained such results.

Multi pass friction stir processing (FSP) plays an important role in refining the grain size. As a result of this refinement, the mechanical properties are improved. The reinforcement of the Al metal matrix with AL_2_O_3_ nanoparticles is considered an influential factor in improving the metal matrix composites (MMCs). Specimens submitted to multi-pass FSP showed an improvement in the tensile strength by 25% more compared to base metal. The enhancement that occurs is due to some factors, some of which include the reinforcement of the metal matrix with ceramic powder during FSP. Moreover, there is good distribution of reinforcement nanoparticles in the composite matrix. The first pass was insufficient to improve the mechanical properties because some voids or defects remained in the matrix. These results are in accordance to the previous results [23,38]. The as-received alloy has the highest elongation. The results reveal that although the addition of Al_2_O_3_ nanopowder increases the strength, it may have decreased ductility as investigated in [39].

### 3.2. Effect of Tool Rotational Speed on Ultimate Tensile Strength (UTS)

**Figure 6:**
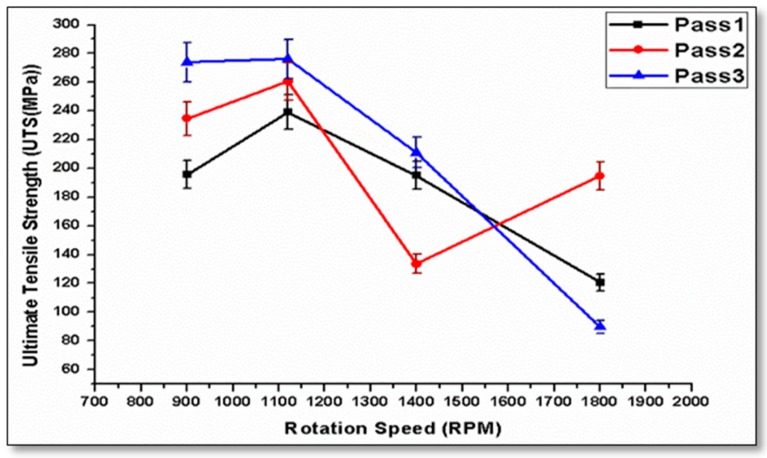
Effect of rotational speed on the ultimate tensile strength at a 10 mm/min travel speed for the three passes. UTS: ultimate tensile strength.

Ultimate tensile strength curves are represented in a comparative form. Figure 6 shows the effect of rotation speed on the ultimate tensile strength throughout different pass numbers. Tool rotation speeds of 900 rpm and 1120 rpm provide a higher ultimate tensile strength as compared to other speeds, especially when processed at 10 mm/min and 14 mm/min traverse speeds. A third pass causes an improvement in the tensile strength. The maximum value for UTS is improved by 27% compared to the base metal. Tool rotation speed at 1800 rpm showed a lower UTS over the three passes performed.

### 3.3. Effect of Tool Traverse Speed on Ultimate Tensile Strength

**Figure 7:**
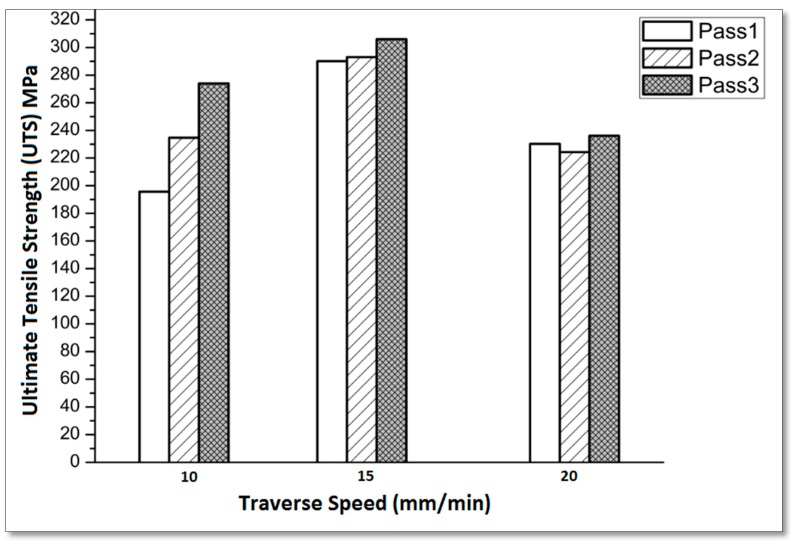
Effect of traverse speed on the maximum tensile strength at a constant rotation speed of 900 rpm for the three passes.

Feed rate also has an effect on the mechanical properties of the Al-alloys. The tool rotation speed with suitable traverse speed constitutes an important parameter in the FSP. Enhanced results for these metal matrix composites are obtained at relatively low speeds with 10 mm/min and 15 mm/min traverse speeds, as shown in Figure 7. Higher traverse speeds used in FSP for the nanocomposite matrix did not generate enough heat during processing. The observed results are consistent with [40,41] showing that increasing the traverse speed decreased the UTS.

### 3.4. Effect of Process Parameters on Young’s Modulus

**Figure 8:**
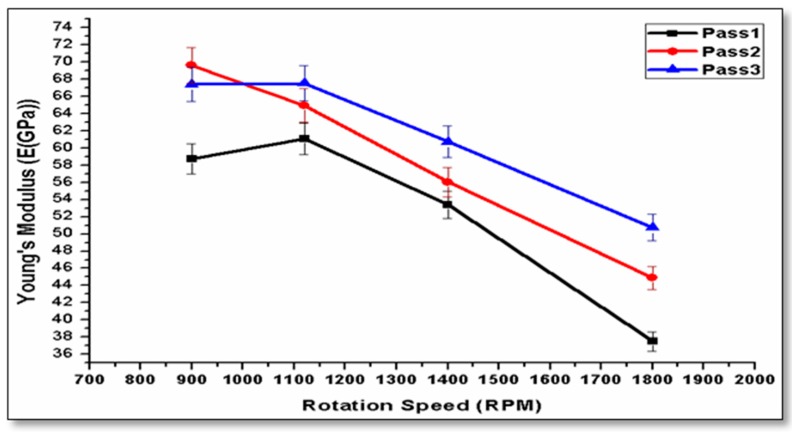
Effect of tool speed on the young’s modulus at 15 mm/min over three passes.

Young’s modulus is considered an important parameter for evaluating mechanical properties. Four samples were conducted for tension testing for each condition in order to obtain the average tensile properties. The measured Young’s modulus is the apparent value, which is estimated from the initial linear portion of the stress–strain curve of the testing machine. The experiments show that increasing the pass number causes an improvement in Young’s modulus, while as the rotation speed increased, the Young’s modulus value decreased. Figure 8 shows the effect of processing parameters on Young’s modulus; the results revealed that Young’s modulus was slightly enhanced, as reported by [21].

### 3.5. Fracture Surface Analysis

**Figure 9:**
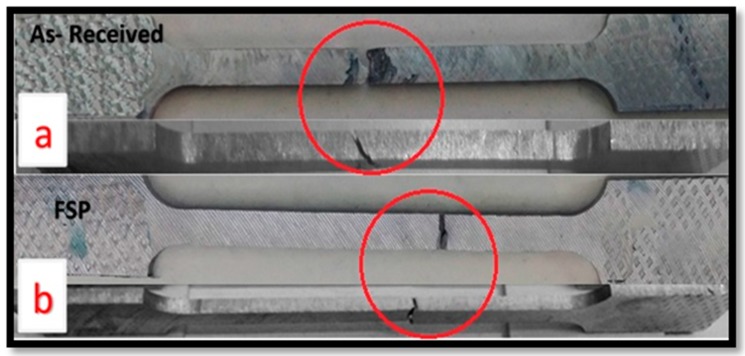
Photograph of the fractured (**a**) as-received and (**b**) friction stir-processed tensile specimens.

**Figure 10:**
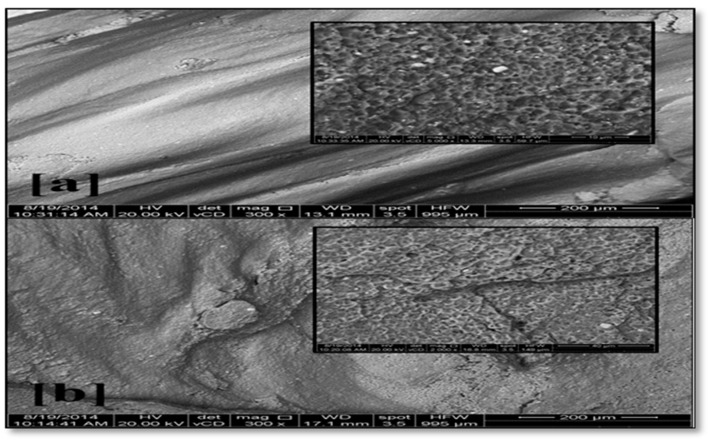
SEM micrograph of fracture zone in AMMNCS specimen friction stir-processed at 1120 rpm at 15 mm/min; (**a**) first pass and (**b**) third pass.

The analysis of the fracture surface of tensile-tested specimens was carried out using a scanning electron microscope (SEM). The macroscopic examination was performed on the fracture surface for friction stirring-processed tensile specimens and the base metal. There was a fracture shape in the base metal AA2024, and the tensile sample appearance showed a 45° angle with the tensile axis. Moreover, there was shear fracture pattern as shown in Figure 9a. The SEM micrographs of fractured surfaces of a single-pass FSP sample processed at 1120 rpm with 15 mm/min traverse speed are shown. The fracture surface of FSP specimens reveals the formation of dimples, which demonstrates a ductile fracture can be observed on the fracture surfaces of the FSP specimens. A straight fracture is noticed on the friction stir-processed specimens as shown in Figure 9b. The analysis represents the two types of brittle fracture—intergranular or trans-granular fracture—by using SEM analysis. The fracture analysis revealed that most of the friction stir-processed samples are characterized as brittle fractures. Figure 10a shows the SEM micrographs of fractured surfaces of single-pass FSP samples processed at 1120 rpm with 15 mm/min traverse speed. The fractured surface of the FSP specimen reveals the formation of dimples, demonstrating that a ductile fracture can be observed on the fracture surfaces of the FSP specimens. Figure 10b demonstrates a brittle fracture in the specimens submitted to third pass FSP due to Al_2_O_3_ nanoparticles and a good distribution of Al_2_Cu intermetallic components. The results are in accordance with [19]. The presence of ceramic particles in the matrix causes a brittle fracture, results consistent with [42].

### 3.6. Nanoparticle Distribution

**Figure 11:**
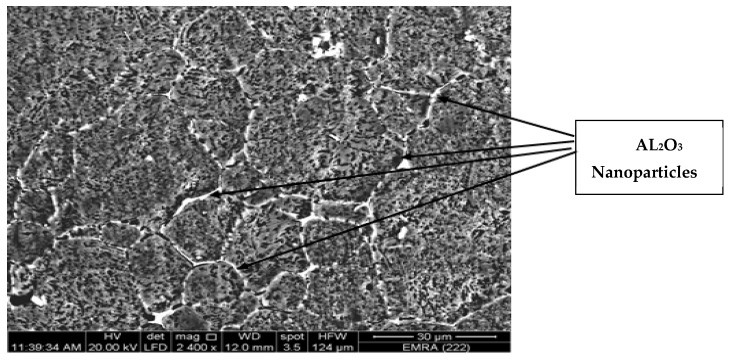
Al_2_O_3_ nanoparticle distribution between the base alloy grain boundaries.

**Figure 12:**
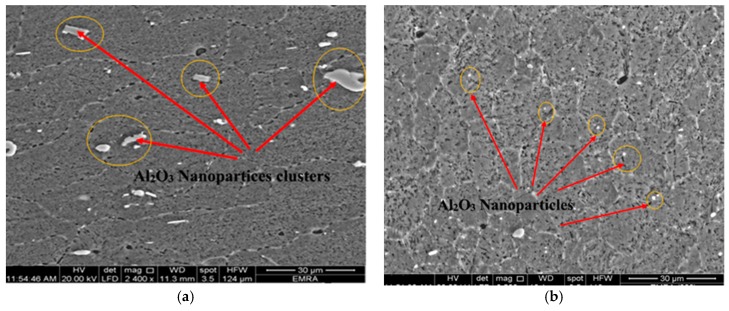
Scanning electron micrograph of Al_2_O_3_ nanoparticle clusters in the composite matrix performed in (**a**) the first pass at 1120 rpm at 15 mm/min; (**b**) the first pass at 1120 rpm at 15 mm/min.

The FSP tool pin rotates at the center line of groove which is filled with AL_2_O_3_ nanoparticles in the base alloy. The stirring action forces the mixture to flow around the tool pin. During traverse tool motion, the materials flow to back of the pin, causing plastic deformation. The mixtures of base alloy and Al_2_O_3_ nanoparticles are exposed to intense breaking and redistribution grains, and thereby Al_2_O_3_ nanoparticles are dispersed in the grain boundaries due to their small size as compared to the size of base alloy granules as illustrated in Figure 11. According to the nature of metal flow in the stirring zone during FSP, large clusters of Al_2_O_3_ nanoparticles are formed at the stirring zone. The specimens underwent the first pass of FSP, leading to brittle fractures in these areas. During the third pass of FSP, the clusters of Al_2_O_3_ nanoparticles can be shattered and well distributed. Multi-pass FSP is considered an effective method to improve the distribution of ceramic nanoparticles in the aluminum metal matrix. Figure 12 shows the effect of pass number on the distribution of nanoparticles during FSP. SEM with EDS analysis illustrates an Al_2_O_3_-map obtained with EDS analysis at the center side of the stir zone. Uniform distribution of Al_2_O_3_ nanoparticles is achieved, and these results are consistent with [26].

**Figure 13:**
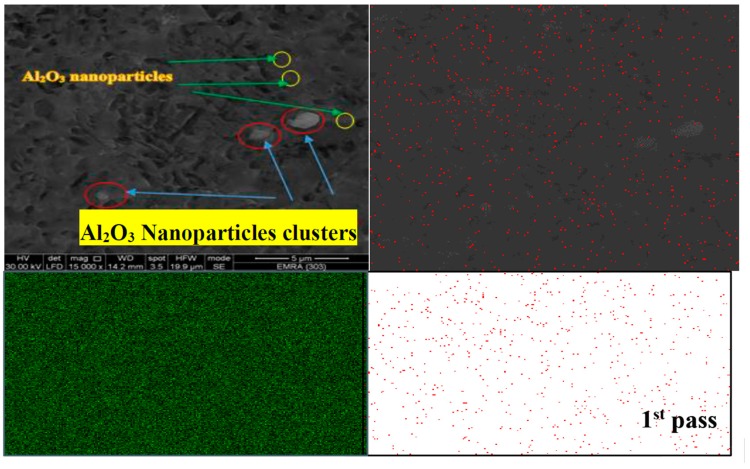
Elemental map of MMNCs and energy-dispersive X-ray spectroscopy (EDS) analysis in FSP at first pass at 1120 rpm at 10 mm/min.

**Figure 14:**
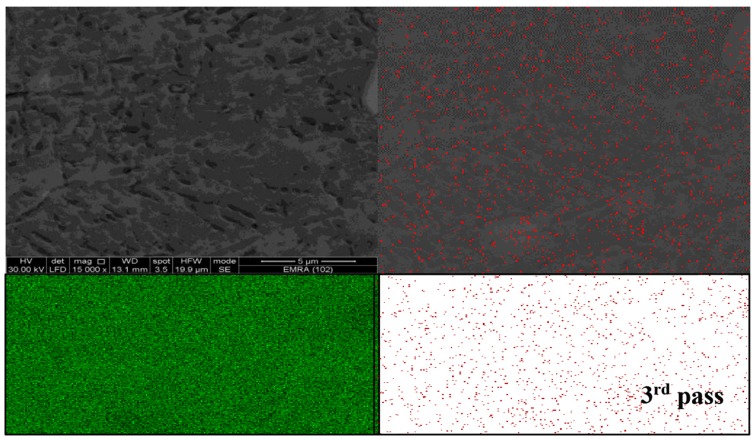
Elemental map of MMNCs and EDS analysis in FSP after the third pass at 1120 rpm at 10 mm/min.

Nanoparticles in both figures appeared in red dots in the EDS analysis. Some clusters formed by Al_2_O_3_ nanoparticles were found in the first pass as shown in Figure 13. After applying the third pass, the accumulated clusters were refined and redistributed again as shown in Figure 14. The analysis confirmed that multi-pass FSP not only achieves a refinement in the grain size but also shows excellent distribution for the reinforcement ceramic powder or nanoparticles additives.

### 3.7. Influence of Tool Speed on the Grain Size

**Figure 15:**
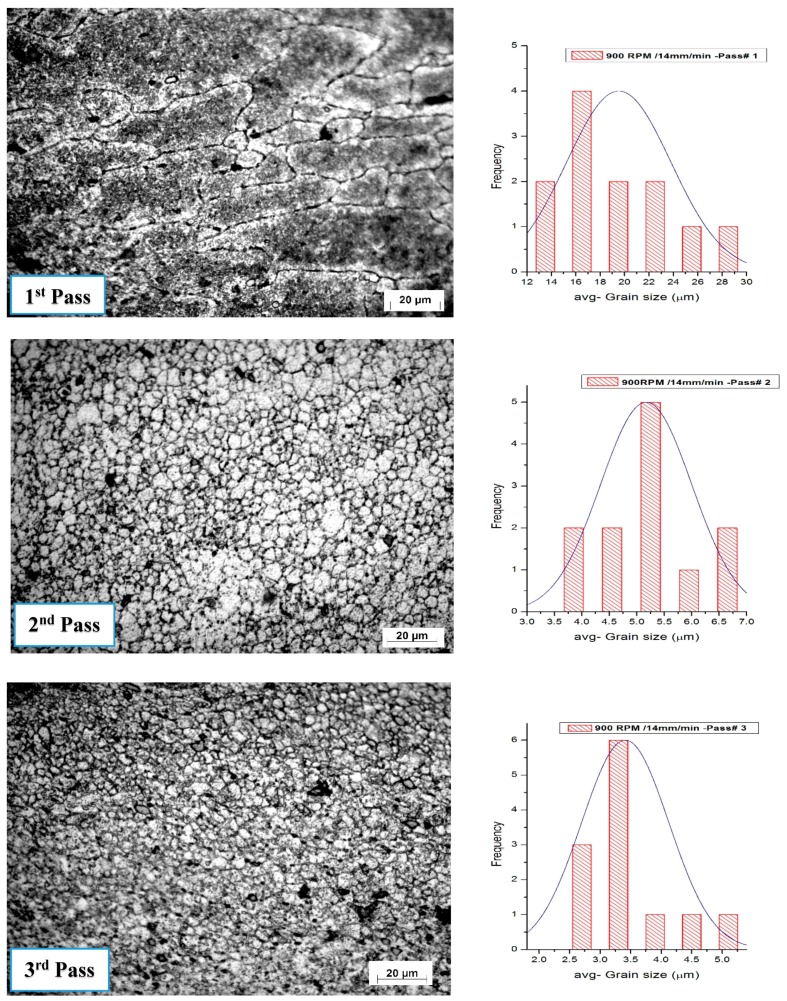
Optical micrographs with histogram showing grain structure and distribution in the stirring zone in friction stir-processed samples at a 900 rpm rotation speed and a 15 mm/min traverse speed.

**Figure 16:**
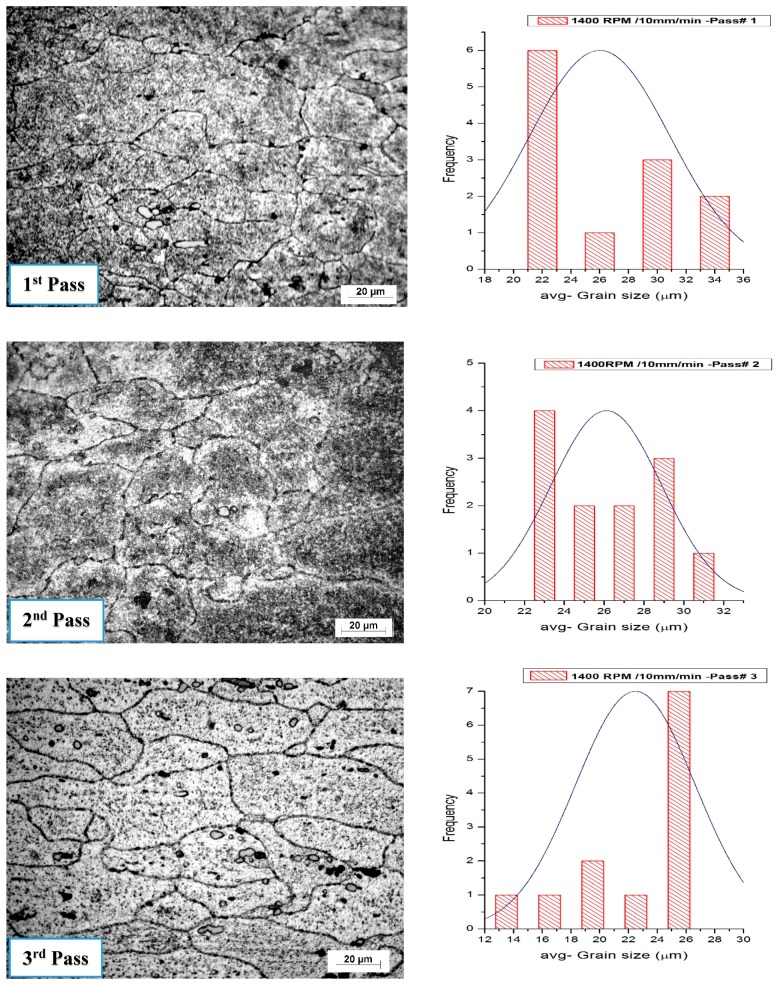
Optical micrographs with histogram showing grain structure and its distribution of stirring zone in friction stir processing at a 1400 rpm rotation speed and a 10 mm/min traverse speed.

The volume fraction of Al_2_O_3_ nanoparticle distribution is enhanced by increasing the pass number. The Al_2_O_3_ nanoparticle volume fraction is calculated using the average grain intercept (AGI) method. The volume fraction has been found in all processing conditions, ranging from 4.7% to 5%. Figure 15 and Figure 16 show the histogram of the grain size distribution. The average grain size for the base alloy is approximately 45 μm, and the aspect ratio is about 31.7%.

**Figure 17:**
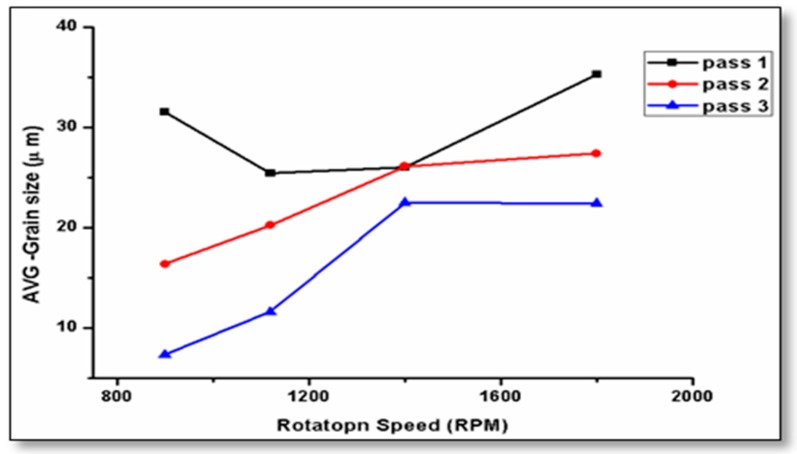
Effect of tool rotation speed on the average grain size, at 10 mm/min traverse speed.

The number of FSP passes has a great effect on the grain structure and the distribution of the precipitates in friction stir-processed samples. The average grain size in the samples was obtained from the stirred zone of specimens. The effect of tool rotation speed on the microstructure grain size is represented in Figure 17. The average grain size decreased through the four different rotation speeds—900, 1120, 1400, and 1800 rpm—processed at a travel speed of 10 mm/min, with the third pass. The tool rotation speed at 900 rpm produces a finer grain. The disparity in the average grain size is obtained at a low rotation speed (900 rpm) and at higher rotation speed (1800 rpm) through all passes. Samples which are carried out at 900 rpm and 10 mm/min travel speed were affected by the multi-pass processing.

### 3.8. Microhardness Results

**Figure 18:**
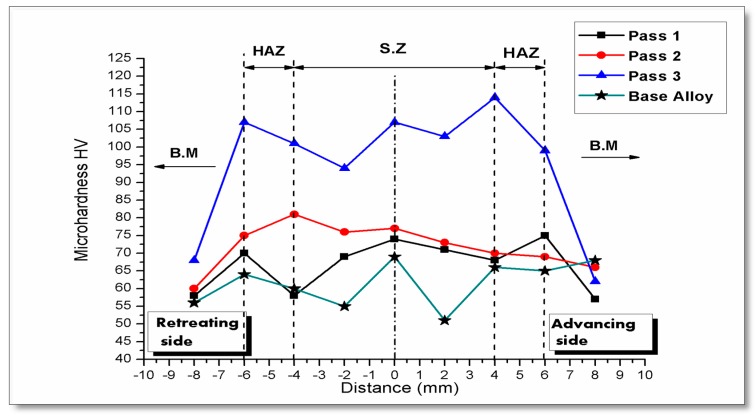
Microhardness profile through a cross section in friction stir processing at 900 rpm with a 10 mm/min traverse speed.

The microhardness results indicated that higher hardness values occurred in the center of the nugget zone (NZ), then gradually decreased across the thermo-mechanically-affected zone (TMAZ) and heat-affected zone (HAZ). The microhardness analysis are focused on the NZ. The processing parameters had a great effect on the hardness of the surface composite. Travelling speed also has an effect on the microhardness results, especially with multi-pass FSP. The hardness increased as the traverse speed increased. The increases in the tool travel speed diminish the effects of heat generated, as well as decrease the exposure time as demonstrated in a travel speed of 20 mm/min at a low rotation speed. Figure 18 shows higher microhardness values for the 10 mm/min travel speed, with lower microhardness results in all processing passes. The results are consistent with those in [43,44]. The average hardness decreased significantly with increased tool rotational speed. This result is intuitive, as the tool speed increased causing the temperature and heat input increases experienced in the processed zone. According to higher temperature, this leads to grain growth, encouraging softening of the material. Furthermore, the increase in temperature maximizes the natural aging effect after processing and leads to a decrease in hardness. The average microhardness results are measured in the stirring zone through four different rotational speeds. The lower microhardness results were obtained at 1400 rpm. The average hardness values which are obtained through all three passes are higher than those of the as-received alloy. The average microhardness value of the base metal AA2024 is 60.2 HV. This is attributed to the high hardness of Al_2_O_3_ nanoparticles and finer microstructure grains. The hardness of SZ increases up to 109.6 HV.

**Figure 19:**
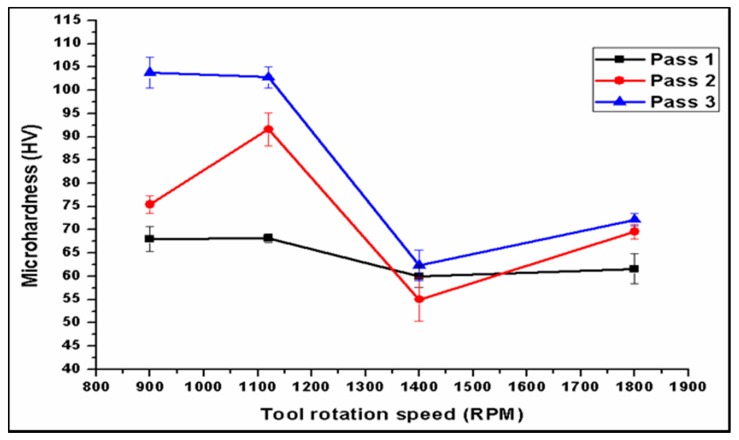
Effect of rotation speed on microhardness through different pass numbers, processed at a 10 mm/s travel speed.

The number of passes played an influential role in the microhardness results, as shown in Figure 19. The results were obtained using a tool rotation speed 900 rpm and a travel speed of 10 mm/min. The average microhardness value in the nugget zone was affected by the pass number, and improved by 46%. Al_2_O_3_ nanoparticles are distributed in friction stir-processed composite, increasing the hardness of the metal matrix. The improvement in the hardness using multi-pass processing is about 49%. By increasing the rotation speed, the relationship between the number of passes and the hardness maintains a direct proportion. Due to a lack of distribution of Al_2_O_3_ nanoparticles in the metal matrix, a rapid stirring of large grains is obtained when using a 1400 rpm rotation speed. The variation between pass numbers gradually decreased and the improvement in the composite matrix also decreased.

The third pass through all of processing speeds resulted in a higher hardness value because of fine grains and good dispersion for Al_2_O_3_ nanoparticles in the matrix. The microhardness results are in accordance with the microstructural observation in the stirring zone; the improvement in the hardness can be attributed to grain size refinement and a better Al_2_O_3_ nanoparticle dispersion in the composite matrix. Multiple friction stir processes have a significant effect on the microhardness results in SZ. The results were obtained at rotation speeds at 900 rpm and 1120 rpm. The lower microhardness results were obtained at 1400 rpm. The average hardness values in first pass, second pass, and third pass friction stir processes of the AA2024/Al_2_O_3_ alloy are higher than those of the as-received alloy. The average microhardness value of the base metal AA2024 is 60.2 HV. This is attributed to the high hardness of Al_2_O_3_ nanoparticles particles and finer microstructure grains. The hardness of SZ increases up to 109.6 HV.

Increasing the rotation speed to 1800 rpm causes a decrease in the microhardness value, especially at low travel speeds of 10 mm/min and 15 mm/min. At higher speeds, the rotation to travel speed (w/v) ratio decreased; an enhanced surface hardness is obtained at the 20 mm/min travel speed. The third pass achieves higher hardness values because of fine grains and good dispersion for Al_2_O_3_ nanoparticles in the matrix. The microhardness results agree with the microstructural observation in the stirring zone. The improvement in the hardness can be attributed to grain size refinement. Pass number has a significant effect on the microhardness results in SZ. The results obtained by rotation speeds at 900 rpm and 1120 rpm are better than the microhardness results at 1400 rpm and 1800 rpm.

## 4. Conclusions

In this study, an attempt has been made to investigate the effects of multi-pass friction stir processing on the surface composite of AA2024/Al_2_O_3_. From this work, several conclusions are derived. The average tensile strength of the friction stirring processed specimen is improved by 10% as compared to the base metal, while the ductility of resultant metal matrix composite was decreased by 50% due to presence of alumina particles in the matrix. Superior tensile strength is achieved at low rotational speeds of 900 rpm and 1120 rpm, with a medium travel speed of 15 mm/min.

Higher rotation speeds cause defects in the composite matrix, principally with higher traverse speeds. Increasing the number of FSP passes leads to finer and more homogenous dispersion of Al_2_O_3_ nanoparticles in the stirring zone. Furthermore, accumulated clusters from reinforcement nanoparticles are significantly reduced. The first pass is not enough to create a metal matrix composite. Therefore, the surface composite layer quality depends on the number of FSP passes.

Multiple-pass FSP enhances the MMNC surface hardness, which goes back to the homogenous distribution of Al_2_O_3_ nanoparticles around the grain boundaries of the base alloy. Average microhardness is increased by 40% in the stirring zone with respect to the as-received alloy, due to grain refinement and reinforcement of nanoparticles.

## Figures and Tables

**Table 1:** Chemical composition of the as-received AA2024 alloy (weight %).

Element	Cu	Mg	Mn	Zn	Fe	Si	Pb
%	4.89	1.45	0.616	0.156	0.11	0.107	0.0193
**Element**	**Ni**	**Cr**	**Sn**	**Ti**	**V**	**Co**	**Al**
%	0.0004	0.00369	0.00792	0.0001	0.00097	0.0004	92.64
